# Efficient Semitransparent Organic Solar Cells Enabled by Ag Grid Electrodes and Optical Coupling Layers

**DOI:** 10.3390/nano13081308

**Published:** 2023-04-07

**Authors:** Ning Zhao, Tao Zhen, Yizhou Wu, Bin Wei, Yingjie Liao, Yuanyuan Liu

**Affiliations:** 1 School of Mechanical Engineering and Automation, Shanghai University, Shanghai 200072, China; 2 Key Laboratory of Advanced Display and System Applications, Ministry of Education, Shanghai University, Shanghai 200072, China

**Keywords:** semitransparent organic solar cells, light utilization efficiency, optical engineering, Ag grid electrode

## Abstract

Semitransparent organic solar cells (ST-OSCs) show great promise for building integrated photovoltaic systems. The balance between power conversion efficiency (PCE) and average visible transmittance (AVT) is a key point of ST-OSCs. We developed a novel semitransparent organic solar cell (ST-OSC) with high PCE and AVT for building integrated renewable energy applications. We used photolithography to fabricate Ag grid bottom electrodes with high figures of merit of 292.46. We also used an optimized active layer of PM6 and Y6, achieving a PCE of 10.65% and an AVT of 22.78% for our ST-OSCs. By adding optical coupling layers of CBP and LiF alternately, we further increased the AVT to 27.61% and the PCE to 10.87%. Importantly, the balance of PCE and AVT can be achieved by the integrated optimization of the active and optical coupling layers, which leads to a significant increase in light utilization efficiency (LUE). These results are of great importance for particle applications of ST-OSCs.

## 1. Introduction

With the development of photovoltaic technology, solar energy is gradually becoming one of the best clean energy sources. Compared with other photovoltaic technologies, organic solar cells (OSCs) have become one of the most promising developments in the photovoltaic industry due to their low cost, light weight, simple fabrication process, and bendability [1,2,3]. With the joint development of molecular design, interface engineering, morphology control, and device structure, the power conversion efficiency (PCE) of OSCs has exceeded 19% [4,5,6]. As an important type of OSC with attractive features of transparency and color tunability, semitransparent organic solar cells (ST-OSCs) demonstrate tremendous potential for building integrated photovoltaics [7,8,9], wearable devices [10], photovoltaic greenhouses [11], and automotive glasses [12,13].

Average visible transmittance (AVT) and PCE are crucial factors in assessing the performance of ST-OSCs. In order to achieve the simultaneous enhancement of AVT and PCE, a large number of studies have concentrated on optimizing the interfacial layer and the photoactive layer. In order to optimize the photoactive layer, the reduction in the donor content can lead to a reduction in the light absorption in the visible region and an increase in the AVT value of ST-OSCs [14,15,16]. For example, Hu et al. prepared ST-OSCs by adjusting the thickness of the active layer, and the PCE and AVT reached 12.37% and 18.6%, respectively [17]. Xue et al. adopted a sequential deposition processing strategy to efficiently prepare ST-OSCs with a PCE of 11.8% and an AVT of 18.6% [16]. By adjusting the ratio of donor to acceptor, the optimal ST-OSC prepared by Hu et al. had a PCE and an AVT of 9.06% and 27.1%, respectively [18]. Furthermore, combining the required optical coupling layer (OCL) with the upper transparent electrode is considered an effective way to improve light absorption while maintaining optical transparency [19,20,21,22]. Li et al. fabricated inverted ST-OSCs with a PCE of 7% and an AVT of 12.2% by combining fine-tuned dielectric mirrors (DMs) [23]. Yu et al. used N pairs of WO_3_/LiF to form a one-dimensional photonic crystal. In the case of N = 8, the PCE of the device with P3HT:ICBA as the active layer reached 4.12%, and the AVT in the 600–800 nm wavelength range was 55.6% [24]. Yeom et al. used the Sb_2_O_3_ optical coupling layer to form the Ag/Sb_2_O_3_/Ag composite electrode. The PCE of the device with PTB7-Th:PC_71_BM as the active layer was as high as 9.71%, and the maximum transmittance was 35.4% [25]. By combining the bilayer dielectric LiF/MoO3, Xu et al. increased the PCE and AVT of the ST-OSC to 13.15% and 25.9%, respectively [26]. All of the above are periodic optical coupling layers that essentially can be categorized as photonic crystals or distributed bragg reflectors. The central wavelength of the photonic crystal is determined according to the absorption range of the active layer material. The basic principle of the design is n_a_d_a_ = n_b_d_b_ = λ_0_/4, where n_a_ and n_b_ represent the refractive index of the optical material, d_a_ and d_b_ represent the thickness of the corresponding optical material, and λ_0_ represents the central wavelength. However, there is no evidence that periodic optical management works better than non-periodic optical management. In a recent report, Xia et al. optimized the thickness of each dielectric layer in the optical coupling layer by an optical simulation model to obtain the optimal nonperiodic optical coupling layer [9]. Liu et al. used the Essential Macleod optical coating design procedure to optimize periodic photonic crystals into non-periodic ones and achieved a higher performance [27]. Therefore, exploring in detail the key balance between photovoltaic performance and optical properties is particularly important in ST-OSCs.

In this paper, we adopt a hybrid modification strategy of donor and acceptor weight ratio adjustment and simple optical coupling layer to simultaneously enhance both the PCE and the AVT. We selected wide bandgap polymer poly[[4,8-bis [5-(2-ethylhexyl)-4-fluoro-2-thienyl]ben-zo [1,2-b:4,5-b′]dithiophene-2,6-diyl]-2,5-thiophenediyl [5,7-bis(2-ethylhexyl)-4,8-dioxo-4H,8H-benzo[1,2-c:4,5-c′]dithiophene-1,3-diyl]-2,5-thiophene diyl] (PM6) as the donor and narrow bandgap small molecule 2,20-((2Z,20Z)-((12,13-bis(2-ethylhexyl)-3,9-diundecyl-12,13-dihydro-[1,2,5]thiadiazolo[3,4-e]thieno-[2,”30′:4′,50]thieno [20,30:4,5]pyrrolo[3,2-g]thieno [20,30:4,5]thieno [3,2-b]indole-2,10-diyl)bis(methanylylidene))bis(5,6-difluoro-3-oxo-2,3-dihydro-1H-indene-2,1-diyldene))dimalononitrile (Y6) as the acceptor. First, the PM6:Y6 active layer weight ratio was optimized to reduce the light absorption in the visible region and increase the device AVT. Then, by combining CBP/LiF/CBP/LiF quadruple OCL, the ST-OSCs further reduced the non-absorption in the visible region and enhanced the near-infrared absorption. Meanwhile, the PCE and AVT of ST-OSCs were improved to 10.87% and 27.61%, respectively.

## 2. Materials and Methods

### 2.1. Materials

PM6 (99%) and Y6 (99%) were purchased from Solarmer Materials Inc. Co., Ltd. (Beijing China) PEDOT: PSS aqueous solutions (Clevios PH1000) were purchased from Xi’an Baolight Co., Ltd. 2-methoxyethanol, 1-chloronaphthalene, zinc acetate dihydrate and ethanolamine were purchased from Macklin (Shanghai China).

### 2.2. Fabrication of Ag Grid Electrodes

**Figure 1:**
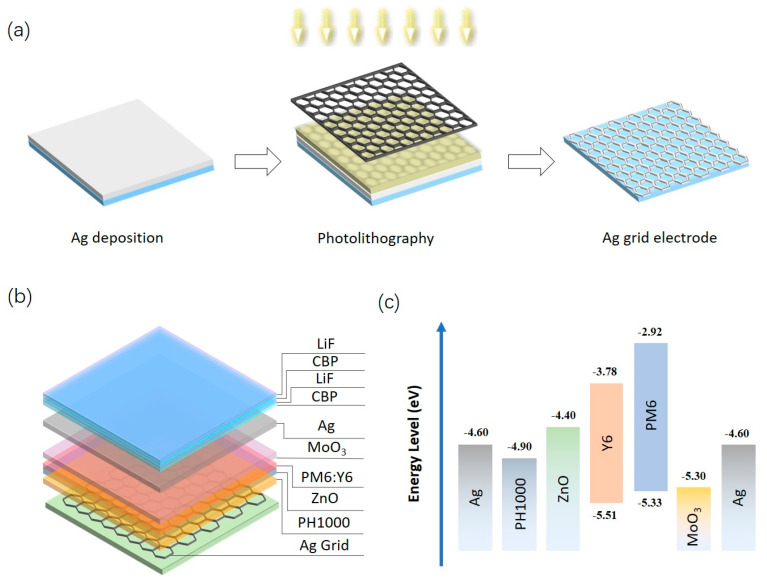
(**a**) Schematic diagram of the manufacturing process of Ag grid electrodes; (**b**) Schematic device structure of the Ag grid ST-OSCs and (**c**) the corresponding energy level diagram.

The fabrication process of Ag grid electrodes is shown in Figure 1a. The glass sub-strates were cleaned sequentially using deionized water, acetone and isopropanol. Sub-sequently, a 50 nm ag film was deposited on the glass substrate. The positive photoresist (AZ GXR-601) was spin-coated onto the ag film at 2000 rpm for 1 min. The photoresist film was heated at 110 °C for 90 s. The photoresist was exposed sequentially using Ul-traμLine 7000 series at an exposure dose of 250 mJ/cm^2^ for 3 s. The exposed photoresist was developed in the developing solution (MF-319) for 30 s. The developed patterned photoresist was bubbled in deionized water to remove the residual developing solution. Next, the patterned photoresist film was baked on a heated plate at 110 °C for 90 s. The patterned ag film was then etched in an ag etching solution (20% wt HNO3) for 30 s to form the patterned ag film. The remaining photoresist and ag etchant were removed by sonication in acetone and deionized water for 2 min to obtain the complete Ag grid electrode.

### 2.3. Fabrication of OSC Devices

PEDOT:PSS (PH1000) was spin-coated on the prepared Ag grid electrode for 60 s at 1000 rpm. The PH1000 layer was annealed at 130 °C for 20 min in air. Deposition at low rotational speeds can produce thick PH1000 layers to flatten the surface and finally prevent shorting of ST-OSCs due to grid surface roughness. The ZnO solution was prepared by mixing 100 mg of zinc acetate dihydrate, 1 mL of methoxyethanol and 30 μL of ethanolamine. This mixture was mechanically stirred at room temperature in a glove box for at least 6 h, and then spin-coated for 30 s at 5000 rpm. The ZnO Layer was annealed at 150 °C for 15 min. When configuring the PM6:Y6 active layer solution with different weight ratios of PM6 to Y6, the dual additives of CN were used, and the volume ratio was 0.5%. The PM6:Y6 layer solution concentration was 15.8 mg mL^−1^. The active layer was spin-coated on substrates in a nitrogen-filled glovebox at 2500 rpm for 30 s to give a thickness of 100 nm. The PM6:Y6 layer was annealed at 110 °C for 10 min. After the deposition of the active layer, 8 nm-thick MoO_3_ and 15-nm thick Ag were deposited by thermal evaporation at 1 × 10^−6^ mbar. The MoO_3_ and ZnO layers were used to increase the exciton dissociation rate. The device structure is Glass/Ag grid/PH1000/ZnO (40 nm)/PM6:Y6 (100 nm)/MoO_3_ (8 nm)/Ag (15 nm), as is shown in Figure 1b. The corresponding energy level diagram is shown in Figure 1c. The four-layers OCL of CBP (40 nm)/LiF (100 nm)/CBP (70 nm)/LiF (40 nm) were deposited on the Ag electrode.

### 2.4. Device Characterization

The Keithley 2400 source meter is adopted to measure the current density versus voltage (J–V) characteristic curve of ST-OSCs. The standard solar simulator (Sun 3000) from the ABET Company provides a standard light source with a light intensity of AM 1.5G (100 mW/cm^2^). The 7-SCSpec solar cell test system from the 7-STAR Co. was used to measure the external quantum efficiencies (EQE). An ultraviolet spectrophotometer (UV-Vis spectrophotometer, U-3900H, Hitachi) was used to characterize the transmittance spectrum, and an atomic force microscope (AFM, Nano Navi SPA-400SPM, Japan) was used to characterize the film morphology. The CIE 1931 color coordinates of ST-OSCs were obtained using a PR-655 spectroradiometer (Photo Research). The film resistance was measured by a four-probes instrument (Four-probe Tester, ST2263, SuZhou China) measurement. The transmission of ST-OSCs with OCL was simulated by the finite-difference time-domain (FDTD) method.

## 3. Results and Discussion

**Figure 2:**
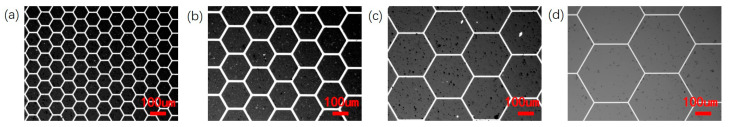
Ag grid electrode images with different pitches (**a**) 100 μm; (**b**) 200 μm; (**c**) 300 μm; (**d**) 400 μm.

Figure 2 shows microscopic images for Ag grid electrodes with a 7 μm line width but different pitches, which are attributed to the mature photolithography process. Different film resistance and transmittance can be obtained by adjusting the pitch of Ag grid electrodes. Table 1 shows that their sheet resistance increases with line pitches, from 14.3 Ω/sq at 100 μm to 66.4 Ω/sq at 400 μm. This trend is reasonable considering the low density of the conducting material at the same deposition thickness and that the line pitches are controlled to 400 μm. The transmittance of Ag grid electrodes also increases with the pitches, from 91% at 100 μm to 97.6% at 400 μm. Based on these results, the optical transmittance and electrical conductivity of Ag grid electrodes are two competing parameters, with the electrical conductivity decreasing when the optical transmittance is increased. For this reason, we introduced the figures of merit (FOM) for a comprehensive evaluation of the optoelectronic properties of the transparent electrode and tried to find an Ag grid parameter with good optical and electrical properties at the same time [28]. The Ag grid electrode with a line width of 7 μm and a pitch of 200 μm shows the highest FOM, which were used as the bottom electrode of the ST-OSCs in this study. Compared with other reported transparent electrodes, the Ag grid electrode exhibits relatively low sheet resistance and high transmittance [28,29,30,31,32].

**Figure 3:**
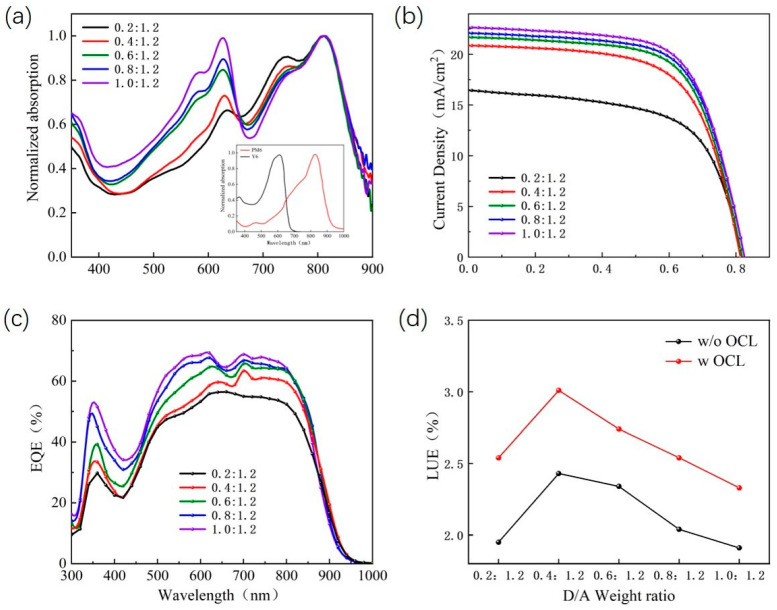
(**a**) The effect of mixed films with different D: A ratios on absorption spectra; Inset: Normalized absorption spectra of PM6 and Y6 films; (**b**) J–V characteristics, (**c**) EQE spectra measured for the ST-OSCs prepared using precursor solutions having different D: A ratios; (**d**) Variation of light utilization efficiency (LUE) in relation to the D: A weight ratio in ST-OSCs with and without OCL.

The inset of Figure 3a shows the UV-Vis spectra of PM6 and Y6 films. Clearly, the photon collection of the pure PM6 film is mainly in the range of 450–700 nm, and the absorption peak is at 620 nm. The pure Y6 films with absorption peaks at 820 nm are mainly collecting photons in the near-infrared range. PM6 and Y6 films have complementary absorption, which facilitates light capture. Figure 3a shows the normalized absorption spectra of different proportions of PM6:Y6 blended films. With the decrease in the PM6 content, the photon capture ability of the active layer in the visible range decreased significantly, and the photon replenishment ability in the near-infrared region increased significantly. The results show that the absorption of the active layer can be greatly improved by adjusting the ratio of the donor to the acceptor. The J–V curves for all ST-OSCs are shown in Figure 3b. Clearly, the value of the short-circuit current density (Jsc) decreases with the decrease in the PM6 content in the active layer of ST-OSCs, which is mainly determined by the photon trapping and phase separation of the active layer. Meanwhile, the open circuit voltage (Voc) value of ST-OSCs decreased slightly with the decrease in the PM6 content. A similar phenomenon was also reported by Hou et al. in PIDTDTQx:PC_70_BM-based OSCs, which may be due to the reduced LUMO-level of acceptors in the active layer [33]. The EQE of the corresponding ST-OSCs device is shown in Figure 3c. It can be seen that when the PM6 content decreases, the EQE spectrum of the device has a more obvious decrease in the range of 400–800 nm than that in the near-infrared range, which is caused by the weakening of the light absorption of the active layer in this wavelength range and is consistent with the test result of the absorption spectrum of the active layer.

Among them, when the weight ratio of PM6 to Y6 is 1.0:1.2, the PCE is the highest at 12.17%, and the AVT is the lowest at 15.78%. The AVT of ST-OSCs can be calculated according to the following equation [33]:

$$AVT=\frac{\int T\left(\lambda \right)V\left(\lambda \right)AM1.5G\left(\lambda \right)d(\lambda )}{\int V\left(\lambda \right)AM1.5G\left(\lambda \right)d(\lambda )}$$

where *T*(*λ*) is the transmission spectrum, *V*(*λ*) is the spectral luminous efficiency function of the human eye, and AM 1.5 (*λ*) is the luminous flux irradiated by AM 1.5 G. It is clear that the two essential parameters of the ST-OSCs, PCE and AVT, need to be compromised. LUE is an excellent parameter to accurately evaluate the comprehensive performance of ST-OSCs. The definition of LUE is as follows [34]:

LUE=PCE∗AVT

The LUE varied with the weight ratio of PM6 to Y6 in the active layer in ST-OSCs, as shown in Figure 3d. Table 2 presents the measured results of J_SC_, V_OC_, the fill factor (FF) and the PCE of ST-OSCs without OCL prepared at different D: A ratios of active layers. These data suggested that the highest LUE of 2.43% was obtained for the ST-OSCs prepared with an active layer with a D: A ratio of 0.4:1.2. The AVT of ST-OSCs prepared from a PM6:Y6 hybrid film with D: A ratio of 0.2:1.2 was 23.43%, which was closest to the benchmark value of 25% [35].

**Figure 4:**
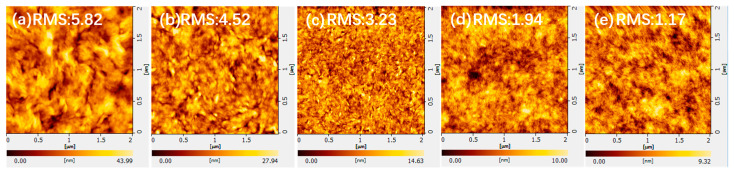
Surface morphology of active layers with different D: A ratios (**a**) 0.2:1.2; (**b**) 0.4:1.2; (**c**) 0.6:1.2; (**d**) 0.8:1.2; (**e**) 1.0:1.2.

One of the necessary conditions for the preparation of highly efficient OSCs is the formation of a favorable surface morphology. We used an AFM to study the surface morphology of active layers with different D: A ratios. It is well known that the degree of phase separation of the active layer plays a crucial role in determining the exciton dissociation, charge transport, and collection [36,37,38]. As is shown in Figure 4, fibrous characteristic structures can be observed in the active layers with different D: A ratios, which is the phenomenon of developing phase separation. When the D: A ratio was varied from 1.0:1.2 to 0.2:1.2, the corresponding root-mean-square (RMS) roughness increased from 1.17 nm to 5.82 nm. These results indicate that with the continuous decrease in PM6 in the active layer, the surface morphology of the PM6:Y6 blended film is negatively affected. This revealed that stronger aggregation of the Y6 acceptor appeared in the active layer as the PM6 content decreased, leading to reduced binding at the donor–acceptor interface [39]. It can be seen that, as the PM6 content decreases, the absorption of the PM6:Y6 hybrid film appears to decrease from 400 nm to 620 nm as well as the higher roughness, which together lead to a reduction in device efficiency.

**Figure 5:**
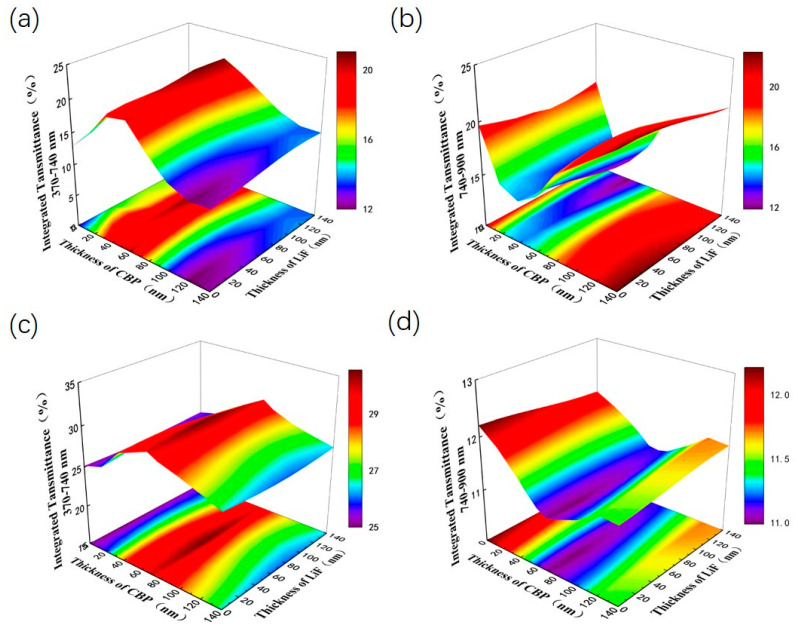
The effects of CBP layer thickness and LiF layer thickness in OCL on the simulated transmittance of ST-OSCs in different wavelength ranges. Double-layers OCL (**a**) 370–740 nm, (**b**) 740–900 nm; Four-layers OCL (**c**) 370–740 nm, (**d**) 740–900 nm.

In terms of optimizing the two competitive indicators of the PCE and AVT, this study employed a high/low dielectric constant structure consisting of CBP and LiF to further optimize the optoelectronic properties of ST-OSCs. The refractive index of the LiF material is 1.39 and the refractive index of the CBP material is 1.697. Thickness combinations of high/low dielectric CBP (40 nm)/LiF (100 nm)/CBP (70 nm)/LiF (40 nm) OCL were optimized for ST-OSCs based on the PM6:Y6 active layer. Figure 5a,b shows the effect of the CBP layer thickness and the LiF layer thickness on the simulated transmittance of ST-OSCs in the wavelength range of 370 to 740 nm and 740 to 900 nm in the CBP/LiF double-layers OCL. Figure 5c,d shows the effect of the CBP layer thickness and LiF layer thickness on the simulated transmittance of ST-OSCs in the wavelength range of 370 to 740 nm and 740 to 900 nm in the latter two layers of the CBP (40 nm)/LiF (100 nm)/CBP/LiF four-layers OCL. Simulations clearly show that the CBP (40 nm)/LiF (100 nm) double-layers OCL has less effect on visible light transmittance and reflects longer wavelength light, while the CBP (40 nm)/LiF (100 nm)/CBP (70 nm)/LiF (40 nm) four-layers OCL can induce distinct optical phenomena, allowing for enhanced visible light transmittance and reflection of long-wavelength light (such as near-infrared light) into the interior of the device. It can be seen that the utilization of a four-layers dielectric helps to improve the visible transmittance and the collection of photons in the near-infrared region.

**Figure 6:**
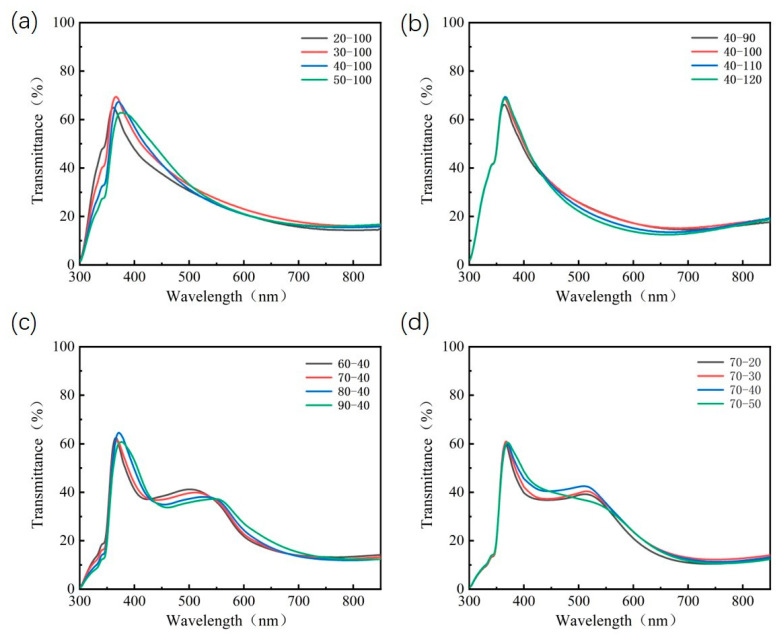
The experimental transmittance spectrum of (**a**) glass/ag (15 nm)/CBP (20–50 nm)/LiF (100 nm); (**b**) glass/ag (15 nm)/CBP (40 nm)/LiF (90–120 nm); (**c**) glass/ag (15 nm)/CBP (40 nm)/LiF (100 nm)/CBP (60–90 nm)/LiF (40 nm); (**d**) glass/ag (15 nm)/CBP (40 nm)/LiF (100 nm)/CBP (70 nm)/LiF (20–40 nm).

We further experimentally validate the simulated results for the ST-OSCs with an OCL. As shown in Figure 6a, when the thickness of the LiF film is kept constant, an increase in the thickness of the CBP layer leads to a red shift in the transmittance spectrum. The AVT reaches a maximum when the CBP thickness is 40 nm and the LIF thickness is 100 nm. As is shown in Figure 6b, the AVT remains essentially constant as the thickness of the LiF film increases. As is shown in Figure 6c,d, in the four-layers OCL, the same red shift is induced with the increase in the CBP layer thickness, while the thickness of the LiF layer has less effect on the AVT. Compared with the samples using double-layers OCL, the samples using four-layers OCL add a new wave peak at around 550 nm, which further improves the AVT. By calculating its AVT, this red-shift phenomenon can effectively increase the optical performance of ST-OSCs. This is consistent with the simulation results above. The structure of glass/ag (15 nm)/CBP (40 nm)/(100 nm)/CBP (70/80 nm)/LiF (40 nm) reached the highest AVT of 39.62%. The transmission spectra of the glass/ag (15 nm)/CBP (40 nm)/LiF (100 nm)/CBP (70 nm)/LiF (40 nm) structure overlap less with the absorption spectrum of the active layer. Therefore, we choose CBP (40 nm)/LiF (100 nm)/CBP (70 nm)/LiF (40 nm) four-layers dielectric as the OCL of ST-OSCs.

**Figure 7:**
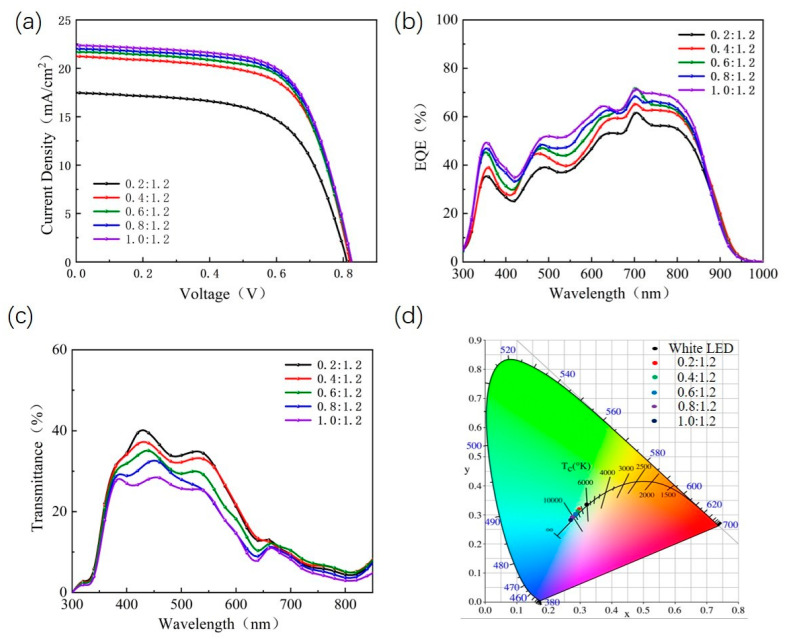
(**a**) J–V characteristics; (**b**) EQE spectrum; (**c**) transmission spectrum; (**d**) CIE (x, y) coordinates measured for the ST-OSCs with OCL prepared using precursor solutions having different D: A ratios.

Figure 7a–c shows the J–V curves, EQE spectra and transmission spectra of ST-OSCs with different D: A ratios. The results show that, as the PM6 content decreases, the photocurrent and EQE subsequently decrease and the AVT is significantly improved. This improvement is mainly due to the weakened absorption of visible light by the ST-OSCs with an OCL, resulting in a lower PCE of the device. It is clear that the performance of the ST-OSCs with an OCL is significantly higher than that of the ST-OSCs without an OCL when the D: A ratio is the same. This improvement is mainly due to the weakened absorption of visible light by the ST-OSCs with an OCL, resulting in a lower PCE of the device. It is additionally clear that the performance of ST-OSCs with an OCL is significantly higher than that of ST-OSCs without an OCL when the D: A ratio is the same. As shown in Figure 3d, when the D: A ratio is 0.4:1.2, the ST-OSCs with an OCL have the highest LUE of 3.01, which is 23.86% higher than that of the ST-OSCs without an OCL. The color perception of ST-OSCs is also a key factor in practical applications besides PCE and AVT, which can be expressed using (x, y) coordinates on the International Committee Éclairage (CIE) chromaticity diagram [33]. As is shown in Figure 7d, the D: A ratio in the active layer increased from 0.2:1.2 to 1.0:1.2, the CIE coordinates of the prepared ST-OSCs changed from (0.2962, 0.3189) to (0.2674, 0.2816) and the corresponding associated color temperature increased from 7628 K to 11912 K. The results show that the lower the PM6 content is in the active layer, the higher the AVT of ST-OSCs is and the closer the CIE (x, y) coordinates are to white light (0.33, 0.34).

**Figure 8:**
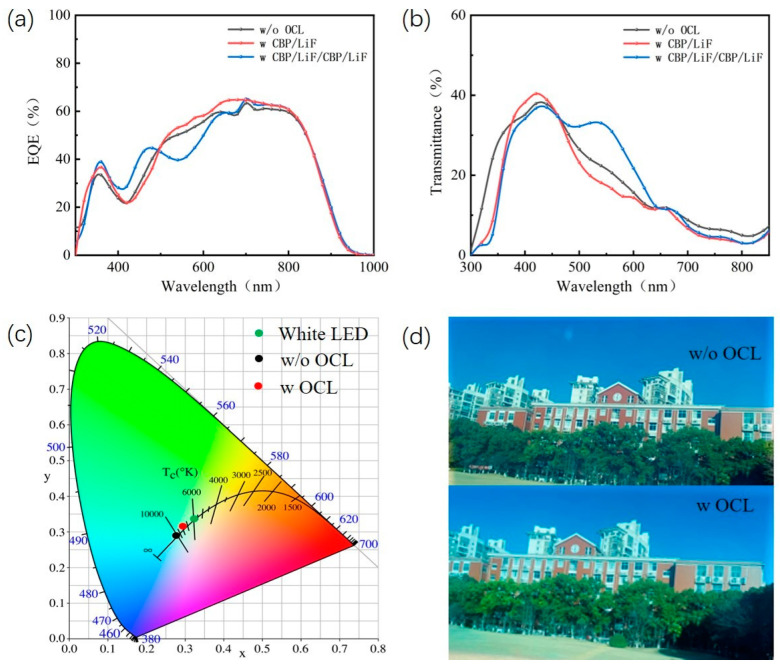
(**a**) The EQE spectra; (**b**) transmission spectra; (**c**) CIE (x, y) coordinates of the ST-OSCs when the weight ratio of PM6 to Y6 is 0.4:1.2. (**d**) Photographs taken through the ST-OSCs with an OCL and without an OCL.

The EQE spectra and transmission spectra of ST-OSCs based on a two-layer OCL and a four-layer OCL are shown in Figure 8a,b. The ST-OSCs with CBP/LiF/CBP/LiF showed a decrease in the range of 450–600 nm and an increase in the near-infrared region in EQE. The ST-OSCs with CBP/LiF showed an increase in EQE in the 450–1000 nm range. The transmission spectra are in agreement with the simulated results. Figure 8c shows the CIE coordinates of ST-OSCs with and without CBP/LiF/CBP/LiF. The results show that the CIE coordinates of the ST-OSCs with the OCL are closer to the white light region, showing good neutral colors. As can be seen in Figure 8d, the picture of ST-OSCs with an OCL is significantly brighter and more natural. It can be clearly seen that the PCE of ST-OSCs can be slightly improved from 10.65 to 10.87% using an optimized OCL compared to ST-OSCs without an OCL. What is impressive is the significant increase in AVT from 22.78 to 27.61% for ST-OSCs with an OCL.

## 4. Conclusions

In summary, we successfully fabricated high-performance ST-OSCs that achieved 10.87% PCE and 27.61% AVT. An Ag grid with a high FOM of 292.46 was made for the bottom electrodes of the ST-OSCs. By reducing the D: A ratio of PM6:Y6 films, the photon collection ability of the active layer is weakened. Optical coupling layers consisting of alternating CBP and LiF were used to further enhance the PCE and AVT of the ST-OSCs. This work shows a useful method for manufacturing high-performance ST-OSCs.

## Figures and Tables

**Table 1:** Parameters for the photovoltaic properties of the Ag grid electrodes.

Pitch (μm)	Sheet Resistance (Ω/sq)	Transmittance (@550 nm%)	FOM
100	14.3	91	273
200	22.9	94.6	292.46
300	36.6	96.3	270.64
400	66.4	97.6	233.7

**Table 2:** Summary of photovoltaic parameters measured for the ST-OSCs prepared using BHJ layers having different weight ratios of PM6 to Y6 with an OCL and without an OCL.

	D:A Ratio	J_SC_ [mA/cm^2^]	V_OC_ [V]	FF [%]	PCE [%]	AVT [%]	LUE [%]
ST-OSCs without OCL	0.2:1.2	16.46 ± 0.24	0.817 ± 0.02	62.10 ± 0.32	8.36 ± 0.21	23.43	1.95
	0.4:1.2	20.65 ± 0.22	0.816 ± 0.03	63.12 ± 0.43	10.65 ± 0.24	22.78	2.43
	0.6:1.2	21.64 ± 0.19	0.818 ± 0.02	65.15 ± 0.25	11.52 ± 0.17	20.37	2.34
	0.8:1.2	22.09 ± 0.29	0.821 ± 0.02	65.23 ± 0.36	11.83 ± 0.07	17.30	2.04
	1.0:1.2	22.65 ± 0.34	0.823 ± 0.01	65.31 ± 0.18	12.17 ± 0.19	15.78	1.91
ST-OSCs with OCL	0.2:1.2	17.45 ± 0.42	0.817 ± 0.02	62.23 ± 0.37	8.7 ± 0.12	28.94	2.54
	0.4:1.2	21.23 ± 0.43	0.816 ± 0.03	63.59 ± 0.34	10.87 ± 0.18	27.61	3.01
	0.6:1.2	21.67 ± 0.32	0.818 ± 0.01	65.17 ± 0.25	11.55 ± 0.22	23.72	2.74
	0.8:1.2	22.01 ± 0.26	0.821 ± 0.02	65.22 ± 0.34	11.76 ± 0.21	21.29	2.54
	1.0:1.2	22.36 ± 0.31	0.823 ± 0.02	65.24 ± 0.36	12.00 ± 0.26	19.43	2.33

## Data Availability

The authors confirm that the data supporting the findings of this study are available within the article.
